# Supplementary material for: Weyl spin-momentum locking in a chiral topological semimetal
Source: Nat Commun. 2024 May 2;15:3720. doi: 10.1038/s41467-024-47976-0 (PMC11066003; doi:10.1038/s41467-024-47976-0)
Supplement: Supplementary file 1 — Supplementary Information [file 41467_2024_47976_MOESM1_ESM.pdf]

Supplementary Information: Weyl spin–momentum locking in a chiral topological semimetal

Jonas A. Krieger,[1,2,*] Samuel Stolz,[3,4,*] Iñigo Robredo,[5,6,*] Kaustuv Manna,[7] Emily C. McFarlane,[1] Mihir Date,[1] Banabir Pal,[1] Jiabao Yang,[1] Eduardo B. Guedes,[8,9] J. Hugo Dil,[8,9] Craig M. Polley,[10] Mats Leandersson,[10] Chandra Shekhar,[5] Horst Borrmann,[5] Qun Yang,[5] Mao Lin,[11] Vladimir N. Strocov,[8] Marco Caputo,[8] Matthew D. Watson,[12] Timur K. Kim,[12] Cephise Cacho,[12] Federico Mazzola,[13,14] Jun Fujii,[15] Ivana Vobornik,[15] Stuart S.P. Parkin,[1] Barry Bradlyn,[11] Claudia Felser,[5] Maia G. Vergniory,[5,6] and Niels B. M. Schröter[1,†]

[1]Max Planck Institut für Mikrostrukturphysik, Weinberg 2, 06120 Halle, Germany
[2]Current address: Laboratory for Muon Spin Spectroscopy, Paul Scherrer Institute, CH-5232 Villigen PSI, Switzerland
[3]Department of Physics, University of California, Berkeley, CA, USA
[4]nanotech@surfaces Laboratory, Empa, Swiss Federal Laboratories for Materials Science and Technology, 8600 Dübendorf, Switzerland
[5]Max Planck Institute for Chemical Physics of Solids, Dresden
[6]Donostia International Physics Center, 20018 Donostia - San Sebastian, Spain
[7]Indian Institute of Technology-Delhi, Hauz Khas, New Delhi 110 016, India
[8]Photon Science Division, Paul Scherrer Institute, 5232 Villigen PSI, Switzerland
[9]Institut de Physique, École Polytechnique Fédérale de Lausanne, 1015 Lausanne, Switzerland
[10]MAX IV Laboratory, Lund University, Fotongatan 2, 22484 Lund, Sweden
[11]Department of Physics, University of Illinois, Urbana-Champaign, USA
[12]Diamond Light Source Ltd, Harwell Science and Innovation Campus, Didcot, OX11 0DE, UK
[13]Istituto Officina dei Materiali, Consiglio Nazionale delle Ricerche, Trieste I-34149, Italy
[14]Department of Molecular Sciences and Nanosystems, Ca' Foscari University of Venice, 30172 Venice, Italy
[15]CNR-IOM, Area Science Park, Strada Statale 14 km 163.5, I-34149 Trieste, Italy

Contents

Supplementary Note 1. Out-of-plane surface state spin-polarization 2
Supplementary Note 2. Removal of noise in the data obtained from the BLOCH beamline 2
Supplementary Note 3. Estimation of systematic errors in spin-polarization of bulk bands shown in Fig. 5 3
Supplementary Note 4. Spin-polarization in dependence of photon energy and polarization of light 3
Supplementary Note 5. Comparison of spin-polarization between two PtGa enantiomorphs 3
Supplementary Note 6. Band structure without spin–orbit coupling 4
Supplementary Note 7. Perturbative analysis of Fermi-arc spin texture 5
Supplementary Note 8. Non-universality of Fermi arc spin texture near $\bar{\text{R}}$ 7
Supplementary Note 9. Josephson diode effects enabled by Weyl-type parallel spin–momentum locking 8
Supplementary Note 10. XPS analysis of surface composition after surface preparation 10
Supplementary References 10

*These authors contributed equally
†Electronic address: niels.schroeter@mpi-halle.mpg.de

Supplementary Note 1. OUT-OF-PLANE SURFACE STATE SPIN-POLARIZATION

In contrast to the omnipresent in-plane spin-polarization, none of our spin-ARPES experiments showed any significant out-of-plane spin-polarization, as shown in Figure 1.

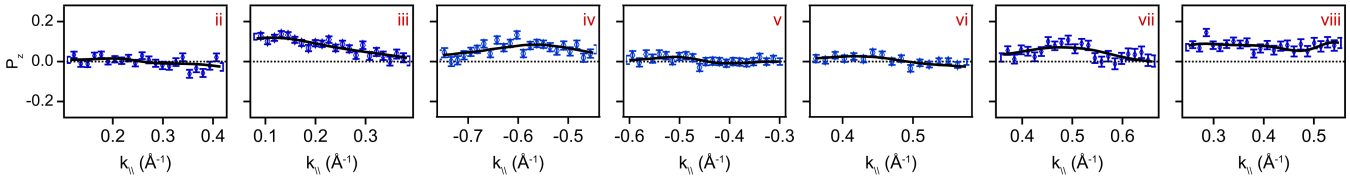

Supplementary Figure 1: **Out-of-plane spin polarization** Asymmetries in the out-of-plane spin-polarizations, corresponding to the cuts in Fig. 3(e) of the main text. The error bars show the statistical error due to counting statistics.

This can be understood when considering the different terminations of PtGa(001). There are two thermodynamically equivalent terminations, related by a 180° roto-translation as shown in Supplementary Figure 2 due to the screw symmetry axes along the principal directions in space group 198. We expect an approximately equal amount of domains from both planes to be present on our (001) polished crystal surfaces, as has been observed in the isostructural PdGa with STM [1]. While the in-plane spin texture is the same in both domains, the out-of-plane texture is opposite (Supplementary Figure 2). Since the ARPES beamspots were large compared to the expected domain size (tens of nm in PdGa [1]), the photoemission signal will be the sum of the signal from both domains, and therefore have a vanishing out-of-plane spin polarization.

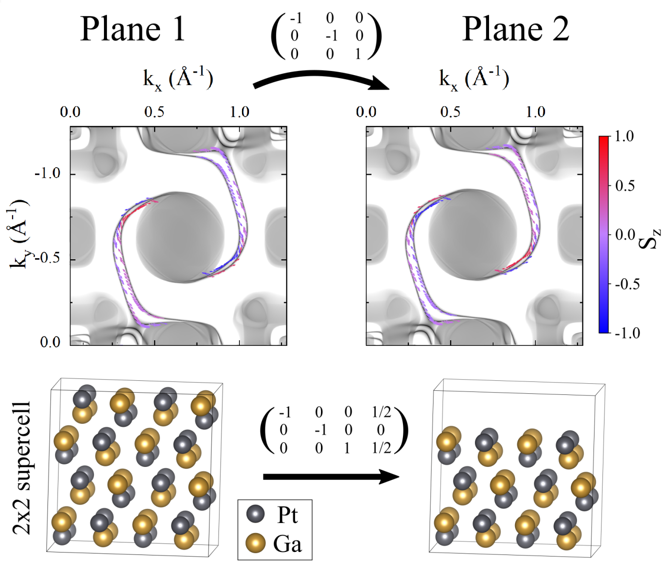

Supplementary Figure 2: **Canceling out-of-plane surface spin polarization** There are two different thermodynamically equivalent cleavage planes, related by a 180° roto-translation (bottom). The corresponding surface spin textures (top) have the same dispersion and in-plane spin texture, but opposite out-of-plane spin texture.

This can result in magnitudes of the measured Fermi-arc spin polarization smaller than 1. Note that the assumption of a polarization magnitude 1 for fitting the data has little effect on the direction of the resulting spin polarizations, but prevents the fit from being overparametrized.

Supplementary Note 2. REMOVAL OF NOISE IN THE DATA OBTAINED FROM THE BLOCH BEAMLINE

Infrequent noise from the preamplifier was removed using the following consideration: Each spectrum consists of an integer multiple of eight scans with different coil polarities, acquired in a $[-++--++-]$ sequence. Within such

a set the highest value N_{max} was flagged as noise if it was more than eleven sigma higher than the second highest value N_{max-1} independent of their respective coil polarities, i.e.

$$N_{max} > N_{max-1} + 11 * \sqrt{N_{max} + 10}. \quad \text{(Supplementary Equation 1)}$$

Values flagged as noise were replaced by 0. In order to not affect the resulting asymmetry, the m other values of opposite polarity were reduced with a scaling factor $(m-1)/m$. This correction was applied to a total of twelve values.

Supplementary Note 3. ESTIMATION OF SYSTEMATIC ERRORS IN SPIN-POLARIZATION OF BULK BANDS SHOWN IN FIG. 5

The asymmetry $A_{c,r}$ for opposite coil polarities was measured for different spin rotation directions $r \in [+,-]$ and coil magnetization directions $c \in [1,2]$. The spin-polarization along the momentum directions was then extracted as

$$P_X = \frac{A_{2,+} - A_{2,-}}{\sqrt{2}S} \quad \text{(Supplementary Equation 2)}$$

$$P_Y = -\frac{A_{2,-} + A_{2,+}}{\sqrt{2}S} \quad \text{(Supplementary Equation 3)}$$

$$P_Z = \frac{A_{1,\pm}}{S}, \quad \text{(Supplementary Equation 4)}$$

where $S = 0.29$ is the Shermann function. We note that this results in right-handed coordinate systems for $\mathbf{k}$ and $\mathbf{P}$ with the Y-direction being vertical and the Z-direction pointing out of the sample surface.

For each setting, a spin polarized background was estimated as the weighted average within a 100 meV wide reference region E_{ref} at the expected bulk band gap, using the propagated counting error (i.e. $\sqrt{N+1}$ for N electrons detected by the channeltron) as a weight .

The spin directions on the band of interest are then extracted on a weighted arithmetic average of the polarization $P_{(d),\xi}$, $\xi \in [X, Y, Z]$ within an energy window $E_{(\text{d})}$; corresponding to the binding energy of the lower branch of the R-point multifold fermion. The choice of where to define the background level E_{ref} introduces a systematical error to the analysis that is significant especially in those cases, where the value of the polarization within $E_{(\text{d})}$ is similar to the value within E_{ref}. In order to estimate this systematical error, we have varied E_{ref} from $[-0.35\,\text{eV}, -0.45\,\text{eV}]$ to $[-0.65\,\text{eV}, -0.75\,\text{eV}]$ in steps of 50 meV, resulting in a set of angles and polarization magnitudes for each choice of E_{ref}. The resulting values systematically depend on E_{ref} and often cluster. We use a weighted arithmetic mean as a best estimate for their value and the weighted variance on that mean as an estimate of the systematic variance . Since the previously calculated statistical errors also slightly depend on the choice of E_{ref}, we use their arithmetic mean as an estimate of the standard deviation due to counting errors. We then use the root of the sum of the squares of these two errors as an upper bound of the combined statistical and systematical error. This quantity is shown as an errorbar in Fig. 5d in the main text.

Supplementary Note 4. SPIN-POLARIZATION IN DEPENDENCE OF PHOTON ENERGY AND POLARIZATION OF LIGHT

To corroborate that the measured spin texture is correctly reflecting the initial state spin polarization, we recorded several momentum distribution curves (MDCs) at different photon energies or light polarization, as shown in Supplementary Figure 3. We find that the main spin polarization is always in the same channel, independent of the photon energy and light polarization used for the spin-ARPES experiments. We can therefore conclude that matrix element effects only play a minor role and that the measured spin texture reflects the spin texture of the initial state.

Supplementary Note 5. COMPARISON OF SPIN-POLARIZATION BETWEEN TWO PTGA ENANTIOMORPHS

The two enantiomorphs of PtGa are related by a mirror operation. The spin polarization behaves under such a mirror as a pseudo-vector, which we confirm by a measurement on the opposite enantomer, shown in Supplementary Figure 4.

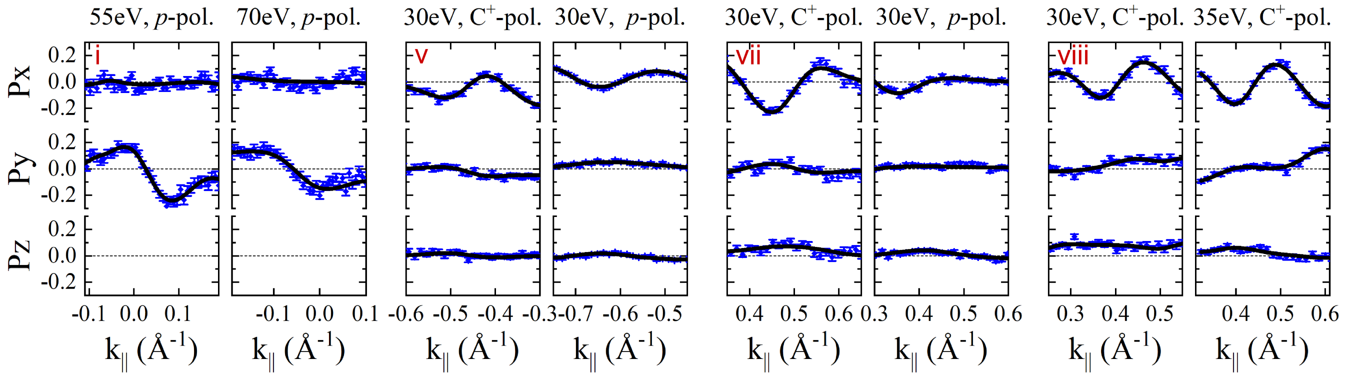

Supplementary Figure 3: **Polarization and photon-energy dependence** Selected spin cuts of Fig. 3(e) from the main text, measured with different photon energies and polarizations. The error bars show the statistical error due to counting statistics.

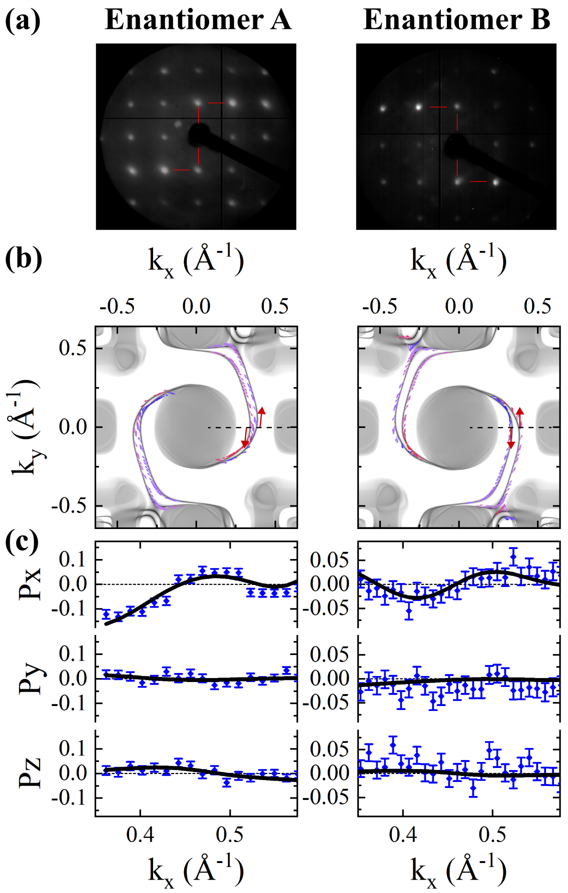

Supplementary Figure 4: **Spin polarization in different enantiomers** (a) LEED images at 97.5 eV showing the different chiralities. (b) Calculated spin textures. (c) Asymmetries in the measured spin-polarisations at 30 eV with C+ polarisation in both enantiomers. The resulting spin directions are shown as red arrows in (b) The dispersions are mirrored, and the spin undergoes a pseudo-vector mirror. The error bars show the statistical error due to counting statistics.

Supplementary Note 6. BAND STRUCTURE WITHOUT SPIN–ORBIT COUPLING

A reference bulk band structure of PtGa without SOC is shown in Supplementary Figure 5

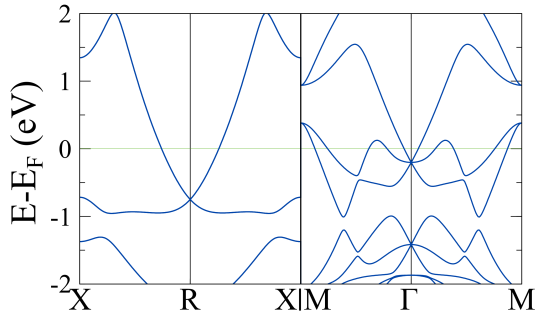

Supplementary Figure 5: **Band structure without SOC** Ab-initio calculated bandstructure without SOC along the same high symmetry directions shown in Fig. 2 of the main text.

Supplementary Note 7. PERTURBATIVE ANALYSIS OF FERMI-ARC SPIN TEXTURE

In this section, we will analyze the $\mathbf{k}\cdot\mathbf{p}$ Hamiltonian for electrons near the Γ point of PtGa for weak spin–orbit coupling, in order to derive the relationship between the bulk and Fermi arc spin textures. As we see in Supplementary Figure 5, without SOC the electronic states in PtGa near the Fermi level at Γ are sixfold degenerate (three spin-degenerate states). The most general symmetry-allowed $\mathbf{k}\cdot\mathbf{p}$ Hamiltonian for these states in the absence of spin–orbit coupling reads [2, 3]

$$H_0(\mathbf{k}) = \epsilon_0 \mathbb{I}_6 + v_{\mathrm{F}}\mathbf{k}\cdot\left(\vec{L}\otimes\sigma_0\right), \qquad \text{(Supplementary Equation 5)}$$

where ϵ_0 is the energy of the sixfold degenerate states at Γ, $\mathbb{I}_6$ is the 6×6 identity matrix, σ_0 is the identity matrix acting on spin, and $\vec{L}$ is a vector of time-reversal odd $\ell = 1$ angular momentum generators that act in the pseudospin space of the threefold degeneracy. They are given explicitly by

$$L_x = \begin{pmatrix} 0 & 0 & 0 \\ 0 & 0 & -i \\ 0 & i & 0 \end{pmatrix}, \quad L_y = \begin{pmatrix} 0 & 0 & i \\ 0 & 0 & 0 \\ i & 0 & 0 \end{pmatrix}, \quad L_z = \begin{pmatrix} 0 & -i & 0 \\ i & 0 & 0 \\ 0 & 0 & 0 \end{pmatrix}. \qquad \text{(Supplementary Equation 6)}$$

The Hamiltonian Supplementary Equation (5) describes three spin-degenerate bands that disperse linearly from Γ, and has eigenvalues

$$\begin{aligned} E_{\pm 1}(\mathbf{k}) &= \epsilon_0 \pm |\mathbf{k}|, \\ E_0(\mathbf{k}) &= \epsilon_0. \end{aligned} \qquad \text{(Supplementary Equation 7)}$$

We can exploit the emergent continuous rotational symmetry of Supplementary Equation (5) to find explicit expressions for the eigenstates of the Hamiltonian. Let us introduce the three eigenstates of L_z with pseudospin azimuthal quantum number $m_z \in \{-1, 0, 1\}$ as

$$|m_z = 1\rangle = \frac{1}{\sqrt{2}}\begin{pmatrix} 1 \\ 0 \\ i \end{pmatrix}, \quad |m_z = 0\rangle = \begin{pmatrix} 0 \\ 1 \\ 0 \end{pmatrix}, \quad |m_z = -1\rangle = \frac{1}{\sqrt{2}}\begin{pmatrix} 1 \\ 0 \\ -i \end{pmatrix}. \qquad \text{(Supplementary Equation 8)}$$

In terms of these pseudospin eigenstates, the energy eigenstates of $H_0(\mathbf{k})$ with momentum $\mathbf{k} = |\mathbf{k}|(\sin\theta\cos\phi, \sin\theta\sin\phi, \cos\theta)$ and energy $E_{m_z}(\mathbf{k})$ and spin σ are given by

$$|\mathbf{k} m_z \sigma\rangle = e^{-iL_z\phi} e^{-iL_y\theta} |m_z\rangle \otimes |\sigma\rangle \qquad \text{(Supplementary Equation 9)}$$

The degeneracy at Γ described by $H_0(\mathbf{k})$ is topologically nontrivial; away from Γ Fermi surfaces in each band have Chern number given by $2m_z$. This implies that in a finite geometry, there will be $2|m_z| = 2$ spin-degenerate Fermi arcs emanating from the surface projection of the Fermi surface surrounding the degeneracy (assuming that no Fermi pockets from other points in the BZ project to the same point in the surface BZ). We can derive the pseudospin texture of the Fermi arc states using a Jackiw-Rebbi approach inspired by Ref. [4]. Let us consider a geometry periodic in the x and y directions, where for $z < 0$ we have a system whose low-energy physics is described by

Supplementary Equation (5), and for $z > 0$ we have the vacuum. While a full treatment of this problem requires a regularization of Supplementary Equation (5) and a detailed form of the boundary potential outside the sample, we can focus instead of the region $z < 0$. Anticipating the existence of a Fermi arc surface state, we look for solutions to the Schrodinger equation

$$v_{\rm F}\left(k_xL_x + k_yL_y - i\frac{\partial}{\partial z}\right)\psi(k_x,k_y,z) = (E(k_x,k_y)-\epsilon_0)\psi(k_x,k_y,z) \qquad \text{(Supplementary Equation 10)}$$

that decay into the bulk of the system with

$$\psi(k_x,k_y,z\to-\infty)=0. \qquad \text{(Supplementary Equation 11)}$$

Furthermore, we assume that we have a Fermi arc whose constant energy contour at $\vec{k}=(k_x,k_y)$ is perpendicular to the to the $\hat{n}=(\cos\theta,\sin\theta)$ direction; this means that

$$E(k_x,k_y)=E(\vec{k}\cdot\hat{n})\equiv E(k_n), \qquad \text{(Supplementary Equation 12)}$$

i.e. the velocity of the Fermi arc state is perpendicular to its constant energy contour. Let us take as an ansatz

$$\psi(k_x,k_y,z)=e^{\alpha z}\left|\vec{k}\right\rangle. \qquad \text{(Supplementary Equation 13)}$$

Inserting this into the Schrodinger equation Supplementary Equation (10) we find

$$(E(k_n)-\epsilon_0)\left|\vec{k}\right\rangle = v_{\rm F}(k_xL_x+k_yL_y-i\alpha L_z)\left|\vec{k}\right\rangle \qquad \text{(Supplementary Equation 14)}$$

$$= v_{\rm F}\left(k_nL_n + k_{n^*}L_{n^*} - i\alpha L_z\right)\left|\vec{k}\right\rangle, \qquad \text{(Supplementary Equation 15)}$$

where $k_{n^*}=-k_x\sin\theta+k_y\cos\theta$, $k_n=\vec{k}\cdot\hat{n}$, and similarly $L_n=L_x\cos\theta+L_y\sin\theta$ and $L_{n^*}=-L_x\sin\theta+L_y\cos\theta$. To proceed further, we note that the term in brackets on the right hand side of Supplementary Equation (15) must be independent of k_{n^*} for the equation to be consistent. Using the fact that the matrices $\vec{L}$ obey angular momentum commutation relations, we have that $L_+=L_{n^*}-iL_z$ is a raising operator for L_n, and $L_-=L_{n^*}+iL_z$ is a lowering operator for L_n, we see that we can satisfy Supplementary Equation (15) if $\alpha=|k_{n^*}|$. For $k_{n^*}>0$, we thus have that

$$(E(k_n)-\epsilon_0)\left|\vec{k}\right\rangle = v_{\rm F}\left(|k_{n^*}|L_+ + k_nL_n\right)\left|\vec{k}\right\rangle, \qquad \text{(Supplementary Equation 16)}$$

implying that $\left|\vec{k}\right\rangle = |(k_x,k_y,0),+1\sigma\rangle$ is the $m_z=+1$ eigenstate of $L_n=\vec{L}\cdot(k_x,k_y,0)/|\vec{k}|$. The energy of the Fermi arc is then $E(k_n)-\epsilon_0=v_{\rm F}k_n$. Similarly, if $k_{n^*}<0$, we have that $\left|\vec{k}\right\rangle=|(k_x,k_y,0),-1\sigma\rangle$ is the $m_z=+1$ eigenstate of $-L_n=\vec{L}\cdot(k_x,k_y,0)/|\vec{k}|$. The energy of the Fermi arc is then $E(k_n)-\epsilon_0=-v_{\rm F}k_{n^*}=v_{\rm F}|k_n|$.

To summarize, we see that each Fermi arc surface state is an $m=+1$ eigenstate of $k_xL_x+k_yL_y$, and so the pseudospin texture of the Fermi arc surface states at a point (k_x,k_y) in the surface Brillouin zone is determined by the pseudospin of the highest-energy eigenstates of $H(k_x,k_y,0)$. Although our $\mathbf{k}\cdot\mathbf{p}$ model cannot determine the direction of the Fermi arcs in the surface Brillouin zone, the topological charge of the Hamiltonian $H_0(\mathbf{k})$ in Supplementary Equation (5) along with time-reversal symmetry implies that the surface must contain a time-reversed pair of spin-degenerate Fermi arcs. Our analysis shows that each Fermi arc surface state is an eigenstate of $k_xL_x+k_yL_y$.

Next, let us analyze the effect of spin–orbit coupling on the bulk and surface states. We begin by writing down the most general spin–orbit corrections to the $\mathbf{k}\cdot\mathbf{p}$ Hamiltonian Supplementary Equation (5) consistent with the space group symmetries and to linear order in $\mathbf{k}$. We start by introducing a vector $\vec{\tilde{L}}$ of time-reversal even symmetric matrices [2, 5]

$$\tilde{L}_x=\begin{pmatrix}0&0&0\\0&0&1\\0&1&0\end{pmatrix},\quad \tilde{L}_y=\begin{pmatrix}0&0&1\\0&0&0\\1&0&0\end{pmatrix},\quad \tilde{L}_z=\begin{pmatrix}0&1&0\\1&0&0\\0&0&0\end{pmatrix}. \qquad \text{(Supplementary Equation 17)}$$

To linear order in $\mathbf{k}$, the Hamiltonian including spin–orbit coupling can be parametrized as

$$H(\mathbf{k}) = H_0(\mathbf{k}) + H_{SOC}(\mathbf{k}) \qquad \text{(Supplementary Equation 18)}$$

$$= v_{\rm F}\mathbf{k}\cdot\vec{L} + \lambda_0\vec{L}\cdot\vec{\sigma} + \lambda_1\mathbf{k}\cdot\sigma + \lambda_2\mathbf{k}\cdot(\vec{\tilde{L}}\times\vec{\sigma}) + \lambda_3\Lambda_{ijk}k_i\tilde{L}_j\sigma_k, \qquad \text{(Supplementary Equation 19)}$$

where $\lambda_0, \lambda_1, \lambda_2, \lambda_3$ are spin–orbit coupling strengths, and Λ_{ijk} is a totally symmetric tensor given by the absolute value of the Levi-Civita symbol. The values of the spin–orbit coupling strengths cannot be determined by symmetry alone. However, for most realistic materials we expect $v_F \gg \lambda_1, \lambda_2, \lambda_3$. On the other hand λ_0 has dimensions of energy and therefore cannot be straightforwardly compared to v_F or the other SOC strengths. Instead, λ_0 determines the splitting between the fourfold and twofold degenerate chiral fermions at Γ in the spin–orbit coupled system. By comparing Supplementary Figure 5 to the spin–orbit coupled spectrum in the main text, we see that λ_0 is comparable in magnitude to the energy offset ϵ_0.

We thus expect the spectrum of Supplementary Equation (19) to exhibit three different regimes of behavior as a function of $\mathbf{k}$. First, for $\lambda_i|\mathbf{k}| < v_F|\mathbf{k}| < \lambda_0$ $(i = 1, 2, 3)$ we can ignore λ_i and treat the unperturbed Hamiltonian $H_0(\mathbf{k})$ as a perturbation to the $\mathbf{k}$-independent term $\lambda_0 \vec{L} \cdot \sigma$. This is the regime considered in Ref. [6], although in that reference no assumptions were made about the relative values of v_F and λ_i.

In the second regime of intermediate $\mathbf{k}$, we have $\lambda_i|\mathbf{k}| < \lambda_0 < v_F|\mathbf{k}|$. In this regime, if we are focused on the highest energy bands of H_0 we can neglect the λ_i and treat λ_0 as a perturbation to $H_0 = v_F\mathbf{k} \cdot \mathbf{L}$. Projecting $\lambda_0 \vec{L} \cdot \vec{\sigma}$ into the spin-degenerate upper bands of H_0. We find using Supplementary Equation(9)

$$\langle \mathbf{k} + 1\sigma | \, \lambda_0 \vec{L} \cdot \vec{\sigma} \, | \mathbf{k} + 1\sigma' \rangle = \lambda_0 \hat{\mathbf{k}} \cdot \vec{\sigma}_{\sigma\sigma'}. \qquad \text{(Supplementary Equation 20)}$$

This means that the first-order effect of spin–orbit coupling is to split the $m = +1$ states of H_0 with a radial spin texture. This is consistent with our ab initio calculations of the bulk spin texture.

In this regime, we can also examine the effect of spin–orbit coupling on the Fermi arc states. We can project the bulk spin–orbit Hamiltonian into the basis $|\psi(k_x, k_y)\sigma\rangle$ of spin-degenerate Fermi arcs to find the spin splitting to lowest order. Since the Fermi arc surface states are eigenstates of $L_n = L_x \cos\theta + L_y \sin\theta$, we immediately find that

$$\langle \psi(k_x, k_y)\sigma | \, \lambda_0 \vec{L} \cdot \vec{\sigma} \, | \psi(k_x, k_y)\sigma' \rangle = \lambda_0 \sigma_n = \lambda_0 (\cos\theta\sigma_x + \sin\theta\sigma_y). \qquad \text{(Supplementary Equation 21)}$$

Thus, to lowest order in perturbation theory the Fermi arc states inherit the bulk parallel spin momentum locking, and have spin oriented perpendicular to the arc direction. We expect this result to apply when the surface potential is approximately spin independent, i.e. when Rashba SOC at the surface can be neglected. We note that examining Supplementary Figure 5 compared to the band structure in Fig. 2 of the main text, we see that $v_F|\mathbf{k}_F| \gtrsim \lambda_0$ for states near the Fermi level, so that second-order spin–orbit effects may be non-negligible. However, it can be shown that in the fourfold degenerate basis of $\lambda_0 \vec{L} \cdot \vec{\sigma}$, both $\mathbf{k} \cdot \vec{L}$ and $\mathbf{k} \cdot \vec{\sigma}$ project to the same 4×4 $\mathbf{k} \cdot \mathbf{p}$ Hamiltonian. This implies that, as long as $\lambda_i|\mathbf{k}| \ll v_F|\mathbf{k}|$ and $\lambda_i|\mathbf{k}| \ll \lambda_0$, the highest-energy bulk bands will have radial spin texture regardless of the relationship between $v_F|\mathbf{k}|$ and λ_0. For the Fermi arc surface states, this implies that even for large spin splitting the surface states will inherit an approximately parallel spin texture from the bulk states.

Finally, the third regime occurs for sufficiently large $|\mathbf{k}|$ such that $v_F|\mathbf{k}| > \lambda_i|\mathbf{k}| > \lambda_0$. In this regime, $\mathbf{k}$-dependent spin textures will arise, and simultaneously the linear $\mathbf{k} \cdot \mathbf{p}$ approximation will begin to break down.

We can extract the numerical values of v_F, λ_i (i=0,1,2,3) by fitting the DFT band calculation to the model presented in Supplementary Equation (19). First, we extract λ_0 from the splitting of the fourfold and twofold degenerate chiral fermions at Γ. Solving the model in Supplementary Equation (19) exactly at Γ, we extract that $\Delta = 3\lambda_0 \approx 308\,\text{meV}$, with Δ the energy splitting between the fourfold and twofold fermions. From here we conclude that $\lambda_0 \approx 102\,\text{meV}$. The fit of $v_F, \lambda_1, \lambda_2, \lambda_3$ requires momentum dependence. We computed the bands using the effective TB model in a cubic grid of side length 0.001 (in units of $2\pi/a$, with a the lattice parameter) with a k-mesh of $21 \times 21 \times 21$ k-points. We extract the numerical value of the parameters from a minimum square fit of the model eigenvalues to the DFT bands. Since the minimisation procedure is very sensitive to the initial guess of parameters and we expect $v_F > \lambda_i$ (i=1,2,3), we will first fit v_F to extract a first guess and then perform a full minimisation. We performed a first minimum square fit imposing $\lambda_1 = \lambda_2 = \lambda_3 = 0$, which results in $v_F \approx 2.85\,\text{eVÅ}$. Starting with this value, we then performed a full minimisation and extracted the fitted values for all parameters: $v_F = 2.80\,\text{eVÅ}$, $\lambda_1 = 1.02\,\text{meVÅ}$, $\lambda_2 = -6.38\,\text{meVÅ}$, $\lambda_3 = -116\,\text{meVÅ}$. The Fermi arcs close to $\bar{\Gamma}$ lie within a window of $|\mathbf{k}| \sim 0.1 - 0.2\,\text{Å}^{-1}$. In this region, the energy scales are then $v_F|\mathbf{k}| \approx 280 - 560\,\text{meV}$, $\lambda_0 \approx 102\,\text{meV}$ and $\lambda_3|\mathbf{k}| \approx 11.6 - 23.2\,\text{meV}$. Notice that even if λ_3 is the biggest, the relationship between these quantities matches the second regime, $\lambda_i|\mathbf{k}| < \lambda_0 < v_F|\mathbf{k}|$. Thus, we confirm that the Fermi arcs close to $\bar{\Gamma}$ in PtGa inherit the bulk radial spin polarisation.

Supplementary Note 8. NON-UNIVERSALITY OF FERMI ARC SPIN TEXTURE NEAR $\bar{\text{R}}$

While the $\mathbf{k} \cdot \mathbf{p}$ analysis of Supplementary Section Supplementary Note 7 allowed for an analytical understanding of the Fermi arc spin texture near the surface projection $\bar{\Gamma}$ of the Γ point, the situation near the surface projection of the R point (denoted as $\bar{\text{R}}$) is quite a bit more complicated. First, examining the bulk band structure near R, in

Supplementary Figure 5 and Fig. 2 of the main text, we see that the multifold node at R is significantly further below the Fermi level than the multifold node at Γ. This means that terms beyond linear order in the $\mathbf{k}\cdot\mathbf{p}$ approximation to the Hamiltonian cannot be neglected, as they were near Γ. Furthermore, the node is eightfold degenerate without spin–orbit coupling, meaning that the parameter space of possible spin–orbit coupling terms in the $\mathbf{k}\cdot\mathbf{p}$ Hamiltonian is larger than near the Γ point, which only involved four bands. Finally, and perhaps most significantly, both the R and M points in the bulk Brillouin zone project onto the $\bar{\mathrm{R}}$ point in the surface Brillouin zone. There is a large hole pocket at M at the Fermi level, surrounding a topologically charged fourfold degeneracy. All four topological bands at M cross the Fermi level. The Fermi arcs near $\bar{\mathrm{R}}$ originate not just from bulk states at R, but are also hybridized with Fermi arcs originating from bulk states at M. We thus do not expect the Fermi arcs near $\bar{\mathrm{R}}$ to reflect any universal aspects of the bulk spin texture near R. In particular, we do not expect to observe parallel spin–momentum locking for these surface states.

Supplementary Note 9. JOSEPHSON DIODE EFFECTS ENABLED BY WEYL-TYPE PARALLEL SPIN–MOMENTUM LOCKING

The recent discovery of superconducting diode effects [7] and Josephson diode effects (JDE) [8–13] in materials with Rashba-type spin–momentum locking has attracted significant attention for their potential to lead to novel superconducting electronics. So far, these effects require an external magnetic field or magnetization applied with a component orthogonal to the supercurrent direction ("orthogonal JDE effect"), making the Josephson diode efficiency sensitive to the angle between the magnetic field (or magnetization) and supercurrent direction [8]. This angular dependency is fixed by the orthogonal spin–momentum locking and cannot be tuned by only considering Rashba materials. This is a limitation for applications that aim to use the JDE for low-dissipation detection of magnetic domains and domain walls, especially when the angular dependence of the JDE needs to be tuned based on the magnetic anisotropy of the materials used. This requirement arises, for example, in racetrack devices with magnets having perpendicular magnetic anisotropy. In such cases, achieving a "parallel JDE effect" becomes necessary, where the diode efficiency is highest when the magnetization is always parallel to the supercurrent direction, independent of the crystallographic orientation of the junction.

In the subsequent discussion, we will demonstrate that the realization of an isotropic parallel JDE effect is achievable *exclusively* through a Weyl-type spin texture. Moreover, we will illustrate how a combination of chiral topological semimetals with Rashba materials can be used for precise tuning of the JDE effect, granting the freedom to select the optimal angle between the magnetic field and supercurrent at which the JDE reaches its maximum efficiency. A JDE can occur in systems lacking both inversion and time-reversal symmetry and manifests as a non-reciprocity of the superconducting critical current, which has recently been experimentally observed [7–13]. Multiple potential origins for a JDE are currently being considered [14–23], including finite momentum Cooper pairing arising from the (i) Meissner screening currents, independently of the spin textures [24] and (ii) from the Zeeman effect on a spin-non-degenerate band structure [8, 25–27]. Here, we will discuss the limit where the Zeeman induced JDE effect dominates, which is has recently been observed to occur in materials with large spin–orbit coupling [28], and could therefore also be relevant for materials such as PtGa.

Following the derivations of Refs. [25, 27], we can estimate the effect of the spin texture on the JDE observables. As a simplified example we restrict our discussion to a 2D Hamiltonian with a Weyl-cone-like dispersion and different spin textures, in an in-plane magnetic field,

$$\mathcal{H}_{\mathbf{k}} = v_{\mathrm{F}}\,|\mathbf{k}|\,\hat{\beta}(\hat{\mathbf{k}})\cdot\sigma + \mathbf{B}\cdot\sigma - \mu, \qquad \text{(Supplementary Equation 22)}$$

where $\mathbf{k} = (k_x, k_y)$ is the in-plane momentum, v_{F} is the Fermi velocity, μ is the chemical potential, $\sigma = (\sigma_x, \sigma_y)$ are the spin Pauli matrices, $\hat{\beta}(\hat{\mathbf{k}})$ describes the spin texture, and $\mathbf{B} = (B_x, B_y)$ is the Zeeman energy corresponding to the in-plane magnetic field, and which results in two bands at

$$E^{\pm}_{\mathbf{k}} = -\mu \pm \left|\mathbf{B} + v_{\mathrm{F}}\,|\mathbf{k}|\,\hat{\beta}(\hat{\mathbf{k}})\right|. \qquad \text{(Supplementary Equation 23)}$$

In order to reflect the different spin textures, we choose $\hat{\beta}(\hat{\mathbf{k}}) = \hat{\mathbf{z}}\times\hat{\mathbf{k}}$ for orthogonal, $\hat{\beta}(\hat{\mathbf{k}}) = \hat{\mathbf{k}}$ for parallel, and $\hat{\beta}(\hat{\mathbf{k}}) = (k_x, -k_y)\,/\,|\mathbf{k}|$ as an example for a mixed Dresselhaus-type texture. The corresponding Fermi-surfaces are all circular, but are displaced in different directions in response to the magnetic field, depending on the spin, textures. This is illustrated in Supplementary Figure 6(a) The resulting imbalance in the charge carrier distribution with respect to $\mathbf{k} = \mathbf{0}$, can lead to a finite-momentum cooper pairing, if this system becomes superconducting, e.g. via a proximity effect as observed in Ref. [8]. In the small field limit, the resulting Cooper pair momentum corresponds to the shift

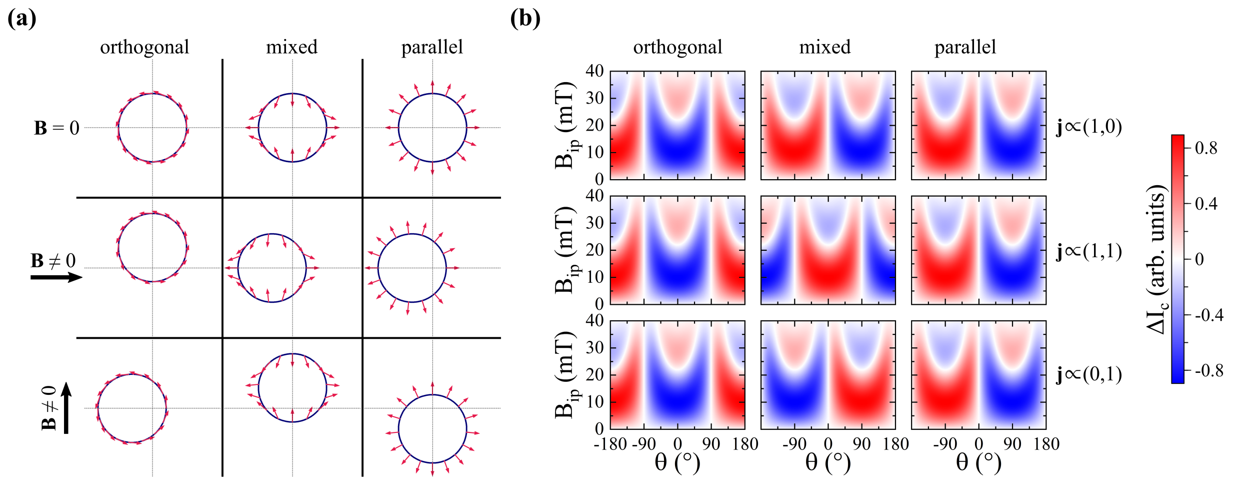

Supplementary Figure 6: **Effect of spin texture on Zeeman induced Josephson Diode Effect (a)** Displacement of Weyl cone Fermi surfaces with different spin textures due to application of a Zeeman field. **(b)** Non-reciprocity in the critical current, resulting from Supplementary Equation (26). Consistently with Ref. [8], we choose $B_c = 45\,\mathrm{mT}$, $B_d = 22\,\mathrm{mT}$ and define the angle θ such that it is 0, ±180 (±90) for a field perpendicular (along) the current and junction direction $\hat{\mathbf{j}}$.

of the Fermi surfaces, as argued in more detail in the Supplementary sections VIII and IX of Ref. [25]. For the three cases (orthogonal, parallel, mixed) different finite momenta are obtained:

$$\mathbf{q}_o = \frac{1}{v_\mathrm{F}}\hat{\mathbf{z}} \times \mathbf{B}, \qquad \mathbf{q}_p = -\frac{1}{v_\mathrm{F}}\mathbf{B}, \qquad \mathbf{q}_m = \frac{1}{v_\mathrm{F}}\begin{pmatrix} -B_x \\ B_y \end{pmatrix}. \qquad \text{(Supplementary Equation 24)}$$

As derived in Eq. 7 of Ref. [8] the resulting non-reciprocity in the critical current ΔI_c due to such a finite momentum pairing $\mathbf{q}$ in a Josephson junction of length d along the current direction $\hat{\mathbf{j}}$, is approximately

$$\Delta I_c \propto \Delta^4 \sin(\delta)\,, \qquad \text{(Supplementary Equation 25)}$$

where $\delta = 2d\mathbf{q}\cdot\hat{\mathbf{j}}$ is the phase shift that the Cooper pair acquires when passing through the junction. The induced superconducting gap $\Delta \propto \sqrt{1-\left(\frac{|\mathbf{B}|}{B_c}\right)^2}$ can be assumed to reflect the field dependence of the order parameter with an in-plane critical field of B_c in the proximitized region. The resulting angular dependence of the JDE strength for different spin textures and directions of the supercurrent is shown in Supplementary Figure 6(b), with the parameters chosen, such that the orthogonal texture reproduces the result of Fig. 2(e,f) from Ref. [8]. Note that analogously to the treatment there, we choose a single material parameter $B_d = \pi v_\mathrm{F}/2d = 22\,\mathrm{mT}$ combining Fermi velocity and junction length, such that

$$\Delta I_\mathrm{c} \propto \left[1-\left(\frac{|\mathbf{B}|}{B_\mathrm{c}}\right)^2\right]^2 \sin\left(\pi\frac{|\mathbf{B}|\,\hat{\mathbf{q}}\cdot\hat{\mathbf{j}}}{B_d}\right) \qquad \text{(Supplementary Equation 26)}$$

Note that from Supplementary Figure 6(b) we see that the resulting JDE strength is independent of the supercurrent direction for the isotropic Rashba- and Weyl-type spin textures. The former realizes the well known "orthogonal" JDE where the maximal ΔI_c can be realized for a field perpendicular to the supercurrent direction $\mathbf{j}$. In contrast, the Weyl-type spin texture realizes a "parallel" JDE where ΔI_c can be maximized for a magnetic field parallel to the current. In contrast, for the mixed Dresselhaus spin texture, the optimal angle varies widely depending on the supercurrent direction.

Recent experiments on the JDE in a heterostructure comprised of a weak link formed from a twisted WTe_2 multilayer have shown that the maximum JDE efficiency is determined by an in-plane magnetic field angle that lies approximately in the center of the two angles expected from the orientation of the top and bottom layers [28]. This suggests that the total phase shift δ_t that the Cooper pairs acquire when tunnelling through the weak link is simply the sum of the phase shift acquired in each layer of the weak link $\delta_t = \delta_1 + \delta_2$. Here δ_1, δ_2 are the phase shifts that the Cooper pairs would obtain if only the top or bottom WTe_2 multilayer was part of the junction.

One can now envision a similar situation (depicted in Supplementary Figure 7) where one combines materials with Rashba- and Weyl-type spin textures to precisely tune the angle between the magnetic field and supercurrent at which the JDE reaches its maximum efficiency.Here we limit ourselves to $\mathbf{j} = \mathbf{j}\hat{\mathbf{x}}$. Analogous to the case of twisted WTe_2, the total phase shift can now assumed to be of the form $\delta_t = \delta_R + \delta_W$, where $\delta_R = 2(B_y d_R/B_R)$, and $\delta_W = 2(B_x d_W/B_W)$ are the phase shifts from the materials with Rashba- and Weyl-type spin textures. Here d_R and d_W are the thicknesses of the two materials, and B_R, B_W are material dependent parameters. Because the thickness of the two materials can potentially be tuned, this enables an effective rescaling of the relative magnitude of the two phase shifts. As a result, the angle between the magnetic field and supercurrent at which the JDE reaches its maximum efficiency can be tuned between the "parallel" and "orthogonal" JDE cases.

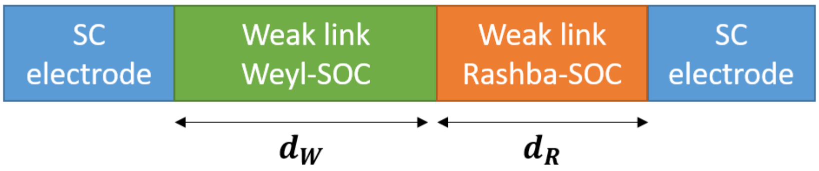

Supplementary Figure 7: Tunable Josephson diode with weak link heterostructure made from two materials with Weyl-type and Rashba-type spin–orbit coupling (SOC) between two superconducting (SC) electrodes.

Supplementary Note 10. XPS ANALYSIS OF SURFACE COMPOSITION AFTER SURFACE PREPARATION

For a rough determination of the elemental surface composition after surface preparation, we performed a fit to our XPS survey spectra, as shown in Supplementary Figure 8. To estimate the atomic ratio we normalize the peak areas by the corresponding calculated atomic photoionization crossection, see Ref. [29]. For the data shown above, this gives a ratio of Pt/Ga≈1, confirming the expected order of magnitude of the atomic ratios of the bulk stoichiometry.

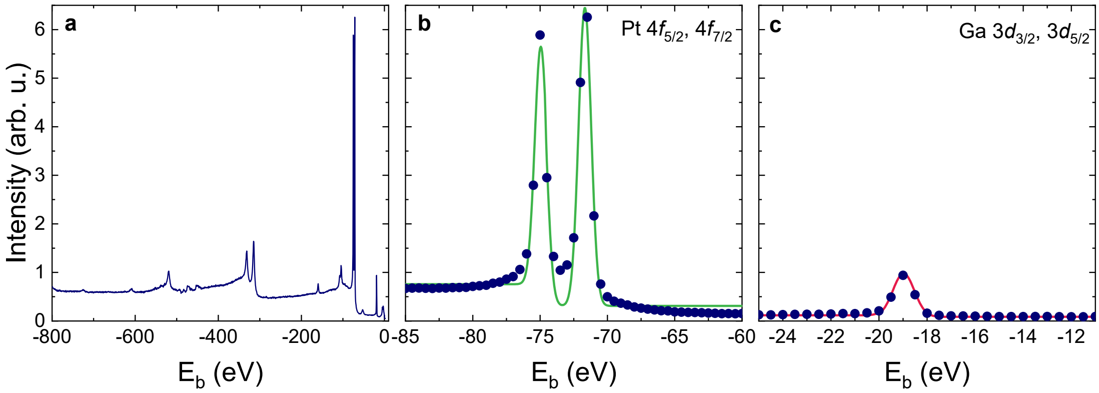

Supplementary Figure 8: **XPS analysis of surface composition (a)** Overview XPS spectra of PtGa 111. **(b,c)** The Pt4f and Ga3d peaks with the solid lines showing the fits used to estimate the atomic ratio.

Supplementary References

[1] Prinz, J. *Surface Science Investigations on Structure and Binding Centers of Intermetallic PdGa Surfaces*. Ph.D. thesis, EPFL, Lausanne (2014). DOI: 10.5075/epfl-thesis-6337.

[2] Bradlyn, B. *et al.* Beyond Dirac and Weyl fermions: Unconventional quasiparticles in conventional crystals. *Science* **353**, aaf5037 (2016).

[3] Manes, J. L. Existence of bulk chiral fermions and crystal symmetry. *Physical Review B* **85**, 155118 (2012).

[4] Okugawa, R. & Murakami, S. Dispersion of Fermi arcs in Weyl semimetals and their evolutions to Dirac cones. *Phys. Rev. B* **89**, 235315 (2014).

[5] Robredo, I. *et al.* Cubic hall viscosity in three-dimensional topological semimetals. *Physical Review Research* **3**, L032068 (2021).
[6] Lin, M. *et al.* Spin-momentum locking from topological quantum chemistry: Applications to multifold fermions. *Physical Review B* **106**, 245101 (2022).
[7] Ando, F. *et al.* Observation of superconducting diode effect. *Nature* **584**, 373–376 (2020).
[8] Pal, B. *et al.* Josephson diode effect from Cooper pair momentum in a topological semimetal. *Nature Physics* **18**, 1228–1233 (2022).
[9] Frattini, N. E. *et al.* 3-wave mixing Josephson dipole element. *Applied Physics Letters* **110**, 222603 (2017).
[10] Bocquillon, E. *et al.* Gapless Andreev bound states in the quantum spin Hall insulator HgTe. *Nature Nanotechnology* **12**, 137–143 (2017).
[11] Baumgartner, C. *et al.* Supercurrent rectification and magnetochiral effects in symmetric Josephson junctions. *Nature Nanotechnology* **17**, 39–44 (2022).
[12] Baumgartner, C. *et al.* Effect of Rashba and Dresselhaus spin–orbit coupling on supercurrent rectification and magnetochiral anisotropy of ballistic Josephson junctions. *Journal of Physics: Condensed Matter* **34**, 154005 (2022).
[13] Wu, H. *et al.* The field-free Josephson diode in a van der Waals heterostructure. *Nature* **604**, 653–656 (2022).
[14] Hu, J., Wu, C. & Dai, X. Proposed Design of a Josephson Diode. *Physical Review Letters* **99**, 067004 (2007).
[15] Zazunov, A., Egger, R., Jonckheere, T. & Martin, T. Anomalous Josephson Current through a Spin-Orbit Coupled Quantum Dot. *Physical Review Letters* **103**, 147004 (2009).
[16] Reynoso, A. A., Usaj, G., Balseiro, C. A., Feinberg, D. & Avignon, M. Spin-orbit-induced chirality of Andreev states in Josephson junctions. *Physical Review B* **86**, 214519 (2012).
[17] Brunetti, A., Zazunov, A., Kundu, A. & Egger, R. Anomalous Josephson current, incipient time-reversal symmetry breaking, and Majorana bound states in interacting multilevel dots. *Physical Review B* **88**, 144515 (2013).
[18] Yokoyama, T., Eto, M. & V. Nazarov, Y. Josephson Current through Semiconductor Nanowire with Spin–Orbit Interaction in Magnetic Field. *Journal of the Physical Society of Japan* **82**, 054703 (2013).
[19] Yokoyama, T., Eto, M. & Nazarov, Y. V. Anomalous Josephson effect induced by spin-orbit interaction and Zeeman effect in semiconductor nanowires. *Physical Review B* **89**, 195407 (2014).
[20] Chen, C.-Z. *et al.* Asymmetric Josephson effect in inversion symmetry breaking topological materials. *Physical Review B* **98**, 075430 (2018).
[21] Pal, S. & Benjamin, C. Quantized Josephson phase battery. *Europhysics Letters* **126**, 57002 (2019).
[22] Kopasov, A. A., Kutlin, A. G. & Mel'nikov, A. S. Geometry controlled superconducting diode and anomalous Josephson effect triggered by the topological phase transition in curved proximitized nanowires. *Physical Review B* **103**, 144520 (2021).
[23] Zhang, Y., Gu, Y., Li, P., Hu, J. & Jiang, K. General Theory of Josephson Diodes. *Physical Review X* **12**, 041013 (2022).
[24] Davydova, M., Prembabu, S. & Fu, L. Universal Josephson diode effect. *Science Advances* **8**, eabo0309 (2022).
[25] Hart, S. *et al.* Controlled finite momentum pairing and spatially varying order parameter in proximitized HgTe quantum wells. *Nature Physics* **13**, 87–93 (2017).
[26] Daido, A., Ikeda, Y. & Yanase, Y. Intrinsic Superconducting Diode Effect. *Physical Review Letters* **128**, 037001 (2022).
[27] Yuan, N. F. Q. & Fu, L. Supercurrent diode effect and finite-momentum superconductors. *Proceedings of the National Academy of Sciences* **119**, e2119548119 (2022).
[28] Kim, J.-K. *et al.* Intrinsic supercurrent non-reciprocity coupled to the crystal structure of a van der Waals Josephson barrier. Preprint at http://arxiv.org/abs/2303.13049 (2023).
[29] Yeh, J. *Atomic Calculation of Photoionization Cross-Section and Asymmetry Parameters* (Gordon and Breach Science Publishers, Langhorne, PE (USA), 1993).
